# Association of Lymphopenia With Risk of Mortality Among Adults in the US General Population

**DOI:** 10.1001/jamanetworkopen.2019.16526

**Published:** 2019-12-02

**Authors:** David A. Zidar, Sadeer G. Al-Kindi, Yongmei Liu, Nikolas I. Krieger, Adam T. Perzynski, Michael Osnard, Christopher Nmai, Donald D. Anthony, Michael M. Lederman, Michael L. Freeman, Robert A. Bonomo, Daniel I. Simon, Jarrod E. Dalton

**Affiliations:** 1 Harrington Heart and Vascular Institute, University Hospitals Cleveland Medical Center, Cleveland, Ohio; 2 Department of Medicine, Louis Stokes Cleveland Veterans Affairs Medical Center, Cleveland, Ohio; 3 School of Medicine, Case Western Reserve University, Cleveland, Ohio; 4 Division of Cardiology, Duke University School of Medicine, Durham, North Carolina; 5 Department of Quantitative Health Sciences, Lerner Research Institute, Cleveland Clinic, Cleveland, Ohio; 6 Center for Healthcare Research and Policy, Case Western Reserve University at MetroHealth, Cleveland, Ohio; 7 Division of Rheumatology, MetroHealth Medical Center, Cleveland, Ohio; 8 Cleveland Clinic Lerner College of Medicine, Case Western Reserve University, Cleveland, Ohio

## Abstract

**Importance:**

Immune dysregulation can increase the risk of infection, malignant neoplasms, and cardiovascular disease, but improved methods are needed to identify and quantify immunologic hazard in the general population.

**Objective:**

To determine whether lymphopenia is associated with reduced survival in outpatients.

**Design, Setting, and Participants:**

This retrospective cohort study of the National Health and Nutrition Examination Survey (NHANES) included participants enrolled from January 1, 1999, to December 31, 2010, a large outpatient sample representative of the US adult population. Associations were evaluated between lymphopenia and other immunohematologic (IH) markers, clinical features, and survival during 12 years of follow-up, completed on December 31, 2011. Spearman correlations, Cox proportional hazards regression models, and Kaplan-Meier curves were used in univariable and multivariable models, allowing for nonlinear associations with bivariate cubic polynomials. Data were analyzed from September 1, 2018, through July 24, 2019.

**Exposures:**

Absolute lymphocyte counts (ALC), red blood cell distribution width (RDW), and C-reactive protein (CRP) level.

**Main Outcomes and Measures:**

All-cause survival.

**Results:**

Among the 31 178 participants, the median (interquartile range) age at baseline was 45 (30-63) years, 16 093 (51.6%) were women, 16 260 (52.2%) were nonwhite, and overall 12-year rate of survival was 82.8%. Relative lymphopenia (≤1500/μL) and severe lymphopenia (≤1000/μL) were observed in 20.1% and 3.0%, respectively, of this general population and were associated with increased risk of mortality (age- and sex-adjusted hazard ratios [HRs], 1.3 [95% CI, 1.2-1.4] and 1.8 [95% CI, 1.6-2.1], respectively) due to cardiovascular and noncardiovascular causes. Lymphopenia was also associated with worse survival in multivariable models, including traditional clinical risk factors, and this risk intensified when accompanied by bone marrow dysregulation (elevated RDW) and/or inflammation (elevated CRP level). Ten-year mortality ranged from 3.8% to 62.1% based on lymphopenia status, tertile of CRP level, and tertile of RDW. A high-risk IH profile was nearly twice as common as type 2 diabetes (19.3% and 10.0% of participants, respectively) and associated with a 3-fold risk of mortality (HR, 3.2; 95% CI, 2.6-4.0). Individuals aged 70 to 79 years with low IH risk had a better 10-year survival (74.1%) than those who were a decade younger with a high-risk IH profile (68.9%).

**Conclusions and Relevance:**

These findings suggest that lymphopenia is associated with reduced survival independently of and additive to traditional risk factors, especially when accompanied by altered erythropoiesis and/or heightened inflammation. Immune risk may be analyzed as a multidimensional entity derived from routine tests, facilitating precision medicine and population health interventions.

## Key Points

**Question:**

Are low lymphocyte levels associated with reduced survival in the general population?

**Findings:**

In this cohort study of 31 178 participants in the 1999 to 2010 National Health and Nutrition Examination Survey, lymphopenia was associated with shortened survival independently of clinical variables, and this risk was further heightened when accompanied by abnormal hematologic (red blood cell distribution width) and/or inflammatory (C-reactive protein) parameters.

**Meaning:**

Based on these findings, patients with lymphopenia, especially those with other immunohematologic abnormalities, may have excess mortality risk; these patients are readily identifiable because tests of lymphocyte levels often occur during routine medical encounters.

## Teaser

This cohort study assesses whether lymphopenia is associated with reduced survival among outpatients enrolled in the US National Health and Nutrition Examination Survey.

## Introduction

Dysregulation of immunologic function is associated with autoimmune disease, infection,^1,2,3^ malignant neoplasms,^4,5,6^ and cardiovascular disease.^7,8,9,10,11^ Inflammation worsens cardiovascular disease outcomes^9,12^ and may promote malignant disease,^13^ whereas immune exhaustion or failure is associated with the pathogenesis of some cancers,^14,15^ sepsis,^16^ and infectious diseases.^17,18^ Drugs to reduce or promote immune activation are increasingly available to treat established disease, but improved methods are needed to test and deploy optimally novel strategies to prevent immune-associated diseases in the general population.

Depending on the clinical context, immune status is typically viewed through the lens of individual measures of immune status. For instance, C- reactive protein (CRP) is a marker of generalized inflammation and improves risk stratification in healthy adults.^19,20^ However, immune pathways often affect multiple variables, some of which are measured routinely as part of primary care. For instance, inflammation can impair erythropoiesis,^21,22^ and an elevated red blood cell distribution width (RDW) is associated with cardiovascular and noncardiovascular illness and death.^23,24,25^ Immune activation can alter T-cell homeostasis through various mechanisms,^26,27,28,29,30,31^ and lymphopenia is among the strongest risk factors for decreased longevity in those with aortic stenosis.^32^ However, to our knowledge, the extent to which lymphopenia is associated with survival in the general population and whether lymphopenia is associated with additive risk beyond previously established risk markers have not been previously studied.

Herein, we characterize the associations among lymphopenia, other readily available inflammatory and hematologic variables, and mortality in a large adult population. We sought to test the hypothesis that lymphopenia may identify a population at enhanced risk, and those with multiple immunohematologic (IH) abnormalities (defined herein as lymphopenia and/or abnormal RDW or CRP levels) may have further reduced longevity.

## Methods

### Data Sources and Study Inclusion Criteria

This retrospective cohort analysis was conducted from September 1, 2018, through July 24, 2019, using data from the first 6 iterations of the continuous National Health and Nutrition Examination Survey (NHANES), collected from January 1, 1999, through December 31, 2010, by the National Center for Health Statistics of the Centers for Disease Control and Prevention. For each iteration of the continuous NHANES, approximately 5000 participants are selected from 15 counties in the United States, creating a sample that represents the noninstitutionalized US population. The protocols of NHANES have been approved by the National Center for Health Statistics ethics review board, and written informed consent was obtained from participants before participation. This analysis was deemed exempt from University Hospitals Cleveland Medical Center institutional review board approval given the use of deidentified publicly available data. This study followed the Strengthening the Reporting of Observational Studies in Epidemiology (STROBE) reporting guideline.^33^

**Figure 1.**
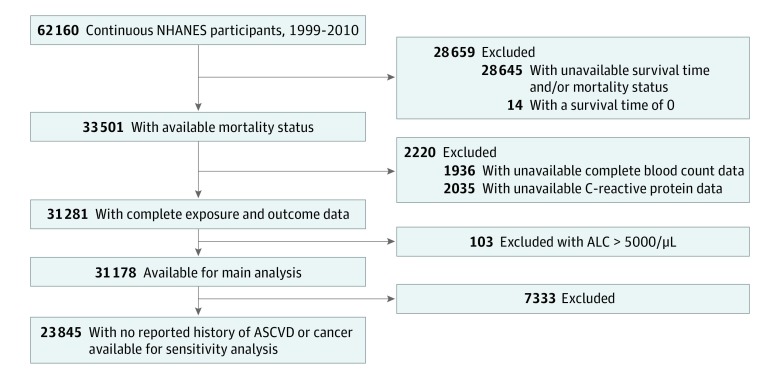
Study Flow Diagram The study population for the main analysis (n = 31 178) consisted of participants continuously enrolled in the 1999-2010 National Health and Nutrition Examination Survey (NHANES) who had complete blood cell count data, no evidence of chronic lymphocytic leukemia (absolute lymphocyte count [ALC]<5000/μL [to convert to 10^9^ per liter, multiply by 0.001]), C-reactive protein levels measured, and survival data. A sensitivity analysis excluded participants with a history of cardiovascular disease or malignant disease (n = 23 845). ASCVD indicates arteriosclerotic cardiovascular disease.

All participants with sufficient identifying data were eligible for linkage with mortality data. Linkage with the National Death Index was performed by the research data center of the National Center for Health Statistics. The National Center for Health Statistics has publicly provided Linked Mortality Files that relate the 1999 to 2010 NHANES participants with National Death Index data. Participants whose mortality data were not available through the Linked Mortality Files were excluded, along with participants with unavailable complete blood cell counts with differentials, participants with an absolute lymphocyte count (ALC) of greater than 5000/μL (to convert to ×10^9^ per liter, multiply by 0.001), and participants with unavailable CRP data. After applying study exclusion criteria, the analytic data set included data from 31 178 NHANES participants (Figure 1), with follow-up completed on December 31, 2011.

### Study Variables

The primary outcome of this study was overall survival associated with study baseline variables. Cause-specific mortality outcomes, namely those resulting from cardiovascular disease, malignant neoplasms, infection, respiratory tract disease, and unintended injuries were studied secondarily. The primary exposure was ALC, and the secondary exposures were RDW and CRP level. Comparative analyses were adjusted for clinical variables prospectively chosen based on their association with survival in the primary prevention setting^34,35^: age at baseline, sex, race/ethnicity, total and high-density lipoprotein cholesterol levels, systolic and diastolic blood pressure readings, type 2 diabetes, and whether or not participants smoked more than 100 cigarettes in their lifetimes.

### Statistical Analysis

Standard numerical and graphical analyses were used to describe distributions of clinical, demographic, and laboratory-based characteristics at baseline in association with levels of ALC, which were defined according to observed quintiles of the distribution. We used Kaplan-Meier analysis to describe univariable associations among common IH and inflammatory biomarkers (ALC, RDW, and CRP level) and clinical risk factors, including type 2 diabetes, age, and time to death.

Our analysis involved measuring the association between ALC and mortality, allowing for heterogeneity in the association according to age at baseline. A multivariable Cox proportional hazards regression model was used to represent these associations in the presence of potential confounding effects associated with the study covariates (described in the Study Variables subsection). In this model, we allowed for nonlinear associations by using bivariate cubic polynomials. We used a Wald χ^2^ test to evaluate the strength of the covariate-adjusted nonlinear interaction effect involving ALC and age (compared with an additive model that included main effects for these variables but no interaction terms). If the interaction was not significant, we then evaluated the main effect of ALC. The resulting model was visualized using covariate-adjusted survival curves. We then studied the sensitivity of the observed ALC effects by deriving an additional Cox proportional hazards regression model that included quintile of RDW or CRP level (in addition to the other study covariates given above).

A secondary analysis explored complex associations with mortality involving age, ALC, RDW, and CRP level, again using multivariable Cox proportional hazards regression. In this model, we used multidimensional (tetravariate) cubic polynomials to represent nonlinear relationships in these 4 dimensions. We adjusted for the same covariates as we did in the primary analysis. We used a Wald χ^2^ test to evaluate the significance of this model against that resulting from the primary analysis (which only included ALC and age). Adjusted 10-year survival was then visualized as a function of age, ALC, RDW, and CRP level based on the resulting model. Another secondary analysis modeled the cause-specific mortality associated with ALC. We used competing-risks survival models, incorporating nonlinear cubic splines, to produce covariate- and competing risks–adjusted hazard ratio (HR) curves as a function of ALC.

Finally, to simplify and to be able to contextualize the aggregate IH risk involving CRP level, RDW, and ALC, we conducted a post hoc analysis in which we developed a simple scoring method. One point was given for having a CRP level or an RDW in the middle tertile and 2 points for having them in the highest tertile. One point was added for having an ALC of greater than 1000/μL and less than 1500/μL and 2 points for an ALC of no greater than 1000/μL.

We used R statistical software, version 3.5.0 (R Project for Statistical Computing) and/or SPSS, version 24 (IBM Corporation) for all analyses. Interaction terms were significant at the level of 2-sided *P* = .10, whereas main effects were significant at the level of *P* = .05.

## Results

### Association of Lymphopenia With Death Due to Cardiovascular Disease, Malignant Neoplasms, and Respiratory Infections

Among the 31 178 participants, the median (interquartile range) age at baseline was 45 (30-63) years; 16 093 (51.6%) were women; 16 260 (52.2%) were nonwhite; and overall 12-year survival was 82.8%. Relative lymphopenia (≤1500/μL) coincided with the lowest quintile (6254 participants [20.1%]) and severe lymphopenia (≤1000/μL) was observed in 937 participants (3.0%). Generally speaking, participants with lymphopenia (lowest vs middle quintile) were older (mean [SD] age, 54.1 [20.6] vs 44.9 [19.2] years), more likely to be male (3418 [54.7%] vs 2653 [47.8%]); non-Hispanic white (3589 [57.4%] vs 2533 [45.6%]), less likely to be of Hispanic ethnicity (1291 [20.6%] vs 1688 [30.4%]), less likely to have asthma (722 [11.6%] vs 666 [12.0%]), and more likely to have anemia (335 [5.4%] vs 165 [3.0%]), congestive heart failure (324 [5.5%] vs 119 [2.4%]), coronary heart disease (400 [6.9%] vs 162 [3.2%]), prior myocardial infarction (403 [6.9%] vs 189 [3.7%]), prior stroke (293 [5.0%] vs 146 [2.9%]), arthritis (1922 [32.8%] vs 1253 [24.9%]), thyroid conditions (624 [10.6%] vs 420 [8.3%]), emphysema (185 [3.2%] vs 85 [1.7%]), and cancer (855 [14.6%] vs 331 [6.6%]) (Table).

**Table.** Demographic and Clinical Parameters According to ALC (N = 31 178)

Variable	% Missing	ALC Quintiles (N = 31 178)				
		300/μL to 1500/μL (n = 6254)	1600/μL to 1900/μL (n = 7889)	2000/μL to 2200/μL (n = 5556)	2300/μL to 2600/μL (n = 5545)	2700/μL to 5000/μL (n = 5934)
Female, No. (%)	NA	2836 (45.3)	3922 (49.7)	2903 (52.2)	3003 (54.2)	3429 (57.8)
Race/ethnicity, No. (%)						
Non-Hispanic white	NA	3589 (57.4)	3933 (49.9)	2533 (45.6)	2396 (43.2)	2467 (41.6)
Hispanic	NA	1291 (20.6)	2149 (27.2)	1688 (30.4)	1786 (32.2)	1889 (31.8)
Non-Hispanic black	NA	1147 (18.3)	1484 (18.8)	1073 (19.3)	1128 (20.3)	1334 (22.5)
Multiple or other	NA	227 (3.6)	323 (4.1)	262 (4.7)	235 (4.2)	244 (4.1)
Age, mean (SD), y	NA	54.1 (20.6)	47.2 (19.7)	44.9 (19.2)	44.1 (18.8)	44.1 (18.1)
Income-to-poverty level ratio, mean (SD)	8.1	2.7 (1.6)	2.6 (1.6)	2.5 (1.6)	2.4 (1.6)	2.3 (1.6)
Red blood cell count, mean (SD), ×10^6^/μL	NA	4.6 (0.5)	4.7 (0.5)	4.7 (0.5)	4.7 (0.5)	4.7 (0.5)
Hematocrit level, mean (SD), %	NA	41.3 (4.7)	41.9 (4.5)	42.0 (4.4)	42.1 (4.4)	42.1 (4.3)
Mean cell hemoglobin level, mean (SD), g/dL	NA	33.9 (0.9)	34.0 (0.9)	34.0 (0.9)	34.0 (0.9)	34.0 (0.9)
ALC, median (IQR), cells/μL	NA	1300 (1200-1400)	1800 (1700-1900)	2100 (2000-2200)	2400 (2300-2500)	3000 (2800-3400)
Absolute monocyte count, median (IQR), cells/μL	NA	500 (400-600)	500 (400-600)	500 (400-600)	600 (500-700)	600 (500-800)
Absolute neutrophil count, median (IQR), cells/μL	NA	3700 (2800-4800)	3900 (3000-5000)	4100 (3200-5200)	4300 (3300-5400)	4600 (3600-5800)
Absolute eosinophil count, median (IQR), cells/μL	NA	100 (100-200)	100 (100-200)	200 (100-200)	200 (100-300)	200 (100-300)
Absolute basophil count, median (IQR), cells/μL	NA	0 (0-0)	0 (0-100)	0 (0-100)	0 (0-100)	100 (0-100)
Red blood cell distribution width, median (IQR), %	NA	12.6 (12.2-13.4)	12.5 (12.1-13.1)	12.5 (12.1-13.1)	12.5 (12.1-13.1)	12.6 (12.2-13.2)
Hemoglobin level, mean (SD), g/dL	NA	14.0 (1.6)	14.2 (1.6)	14.3 (1.5)	14.3 (1.5)	14.3 (1.5)
Platelet count, median (IQR), ×10^3^/μL	NA	233 (196-276)	248 (211-290)	258 (218-300)	267 (229-311)	279 (238-327)
Mean platelet volume, mean (SD), fL	NA	8.1 (0.9)	8.1 (0.9)	8.1 (0.9)	8.1 (0.9)	8.1 (0.9)
Mean corpuscular volume, mean (SD), %	NA	90.3 (5.8)	89.7 (5.5)	89.4 (5.4)	89.2 (5.6)	89.2 (5.5)
Comorbid illness present, No. (%)						
Asthma	0.1	722 (11.6)	966 (12.3)	666 (12.0)	719 (13.0)	823 (13.9)
Anemia	0.1	335 (5.4)	298 (3.8)	165 (3.0)	164 (3.0)	182 (3.1)
Congestive heart failure	8.6	324 (5.5)	188 (2.6)	119 (2.4)	131 (2.6)	140 (2.6)
Coronary heart disease	8.7	400 (6.9)	308 (4.3)	162 (3.2)	186 (3.7)	157 (2.9)
Angina	8.6	272 (4.6)	226 (3.1)	137 (2.7)	129 (2.6)	147 (2.7)
Myocardial infarction	8.5	403 (6.9)	332 (4.6)	189 (3.7)	174 (3.5)	179 (3.3)
Stroke	8.4	293 (5.0)	261 (3.6)	146 (2.9)	151 (3.0)	183 (3.4)
Arthritis	8.4	1922 (32.8)	1855 (25.6)	1253 (24.9)	1175 (23.5)	1368 (25.4)
Thyroid condition	8.5	624 (10.6)	625 (8.6)	420 (8.3)	421 (8.4)	455 (8.4)
Emphysema	8.4	185 (3.2)	140 (1.9)	85 (1.7)	81 (1.6)	103 (1.9)
Chronic bronchitis	8.5	386 (6.6)	384 (5.3)	266 (5.3)	203 (5.8)	364 (6.8)
Liver disease	8.5	218 (3.7)	229 (3.2)	151 (3.0)	160 (3.2)	173 (3.2)
Cancer	8.4	855 (14.6)	655 (9.1)	331 (6.6)	336 (6.7)	353 (6.5)
SBP, median (IQR), mm Hg	4.2	122 (112-138)	120 (110-132)	120 (110-132)	119 (110-132)	120 (112-134)
DBP, median (IQR), mm Hg	4.2	70 (60-76)	70 (62-78)	70 (62-78)	70 (62-78)	70 (62-78)
BMI, median (IQR)	2.0	26.3 (23.2-30.0)	27.0 (23.7-31.0)	27.5 (24.0-31.9)	27.9 (24.2-32.4)	28.7 (24.9-33.4)
Waist circumference, mean (SD), cm	4.0	95.6 (15.0)	96.1 (15.5)	97.1 (16.1)	97.7 (16.2)	99.5 (16.4)
Hemoglobin A_1c_ level, median (IQR), %	0.2	5.4 (5.1-5.7)	5.4 (5.1-5.7)	5.4 (5.1-5.7)	5.4 (5.1-5.7)	5.5 (5.2-5.9)
Albumin level, mean (SD), g/dL	0.7	4.2 (0.4)	4.2 (0.4)	4.3 (0.4)	4.3 (0.4)	4.3 (0.3)
Serum urea nitrogen level, median (IQR), mg/dL	0.7	13 (10-17)	12 (10-16)	12 (9-15)	12 (9-15)	12 (9-15)
Total calcium level, mean (SD), mg/dL	0.7	9.4 (0.4)	9.4 (0.4)	9.5 (0.4)	9.5 (0.4)	9.5 (0.4)
Creatinine level, median (IQR), mg/dL	0.7	0.9 (0.7-1.1)	0.8 (0.7-1.0)	0.8 (0.7-1.0)	0.8 (0.7-1.0)	0.8 (0.7-1.0)
Serum glucose level, median (IQR), mg/dL	0.7	92 (85-102)	91 (84-100)	90 (83-100)	90 (84-100)	90 (83-101)
Total bilirubin level, median (IQR), mg/dL	0.7	0.7 (0.6-0.9)	0.7 (0.5-0.9)	0.7 (0.5-0.8)	0.7 (0.5-0.8)	0.6 (0.5-0.8)
Total cholesterol level, mean (SD), mg/dL	0.5	194 (42)	196 (42)	197 (42)	198 (42)	200 (43)
HDL cholesterol level, median (IQR), mg/dL	0.5	54 (44-66)	52 (43-63)	50 (42-61)	49 (40-59)	47 (39-57)

Abbreviations: ALC, absolute lymphocyte count; BMI, body mass index (calculated as weight in kilograms divided by square of height in meters); DBP, diastolic blood pressure; HDL, high-density lipoprotein; IQR, interquartile range; NA, not applicable; SBP, systolic blood pressure;

SI conversion factors: To convert albumin to grams per liter, multiply by 10; bilirubin to micromoles per liter, multiply by 17.104; calcium to millimoles per liter, multiply by 0.25; cholesterol to millimoles per liter, multiply by 0.0259; creatinine to micromoles per liter, multiply by 88.4; glucose to millimoles per liter, multiply by 0.0555; hemoglobin to grams per liter, multiply by 10.0; hemoglobin A_1c_ to proportion of total hemoglobin, multiply by 0.01; red blood cells to ×10^12^/L, multiply by 1.0; serum urea nitrogen to millimoles per liter, multiply by 0.357; and white blood cells to ×10^9^ per liter, multiply by 0.001.

**Figure 2.**
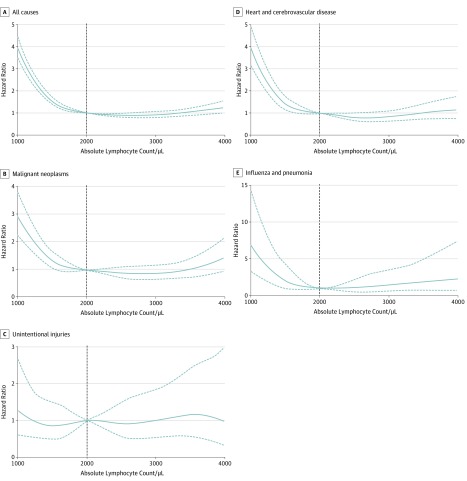
Association of Lymphopenia With Mortality Univariable associations between absolute lymphocyte counts, centered at a count of 2000/μL (vertical dotted line) (to convert to ×10^9^ per liter, multiply by 0.001), and mortality due to all causes, cancer, unintentional injuries (accidents), cardiovascular disease, and influenza and pneumonia. Solid lines indicate hazard ratio point estimates; dotted lines, 95% CI.

The univariable association between ALC and all-cause death increased steeply for ALCs below 2000/μL. For instance, the unadjusted HR for an ALC of 1500/μL was 1.5 (95% CI, 1.4-1.7) and for an ALC of 1000/μL was 3.9 (95% CI, 3.5-4.4). The increased mortality was due to cardiovascular disease (unadjusted HR for ALC of 1500/μL was 1.4 [95% CI, 1.2-1.8]; for ALC of 1000/μL, 4.0 [95% CI, 3.2-5.0]), malignant neoplasm (unadjusted HR for ALC of 1500/μL was 1.3 [95% CI, 1.1-1.7]; for ALC of 1000/μL, 3.0 [95% CI, 2.3-3.9]), and influenza or pneumonia (unadjusted HR for ALC of 1500/μL, 2.1 [95% CI, 1.0-4.3]; for ALC of <1000/μL, 7.0 [95% CI, 3.4-14.7]) but not deaths due to unintentional injuries (unadjusted HR for ALC of <1500 was 0.9 [95% CI, 0.5-1.5]; for ALC of <1000/μL, 1.3 [95% CI, 0.6-2.7]) (Figure 2). In Cox proportional hazards regression models adjusting for age and sex, compared with participants without lymphopenia (ALC >1500/μL; 24 924 [79.9% of the population]), those with mild lymphopenia (ALC 1100/μL to 1500/μL; 5317 [17.1% of the population]) and with severe lymphopenia (ALC ≤1000/μL; 937 [3.0% of the population]) had adjusted HRs of 1.3 (95% CI, 1.2-1.4) and 1.8 (95% CI, 1.6-2.1), respectively.

### Risk Associated With Lymphopenia When Accompanied by Anisocytosis and/or Inflammation

In addition to ALC, Kaplan-Meier estimates show that several other IH biomarkers are individually associated with survival, with RDW and CRP level also exhibiting large survival differences (eFigure 1 in the Supplement). Spearman correlation coefficients and their associated 95% CIs (eTable in the Supplement) indicate generally low degrees of correlation among these common IH biomarkers, with ALC being most closely associated with absolute monocyte count (*r* = 0.30; 95% CI, 0.28-0.31). Red blood cell distribution width exhibited a positive correlation with CRP level (*r* = 0.23; 95% CI, 0.21-0.24), whereas ALC did not closely correlate with RDW (*r* = −0.04; 95% CI, −0.05 to −0.03) or CRP level (*r* = 0.07; 95% CI, 0.06-0.08). Absolute lymphocyte count was also not associated with red blood cell counts, hematocrit level, mean cell hemoglobin concentration, and mean platelet volume, whereas increases in ALC were associated with elevated platelet counts (*r* = 0.25; 95% CI, 0.24-0.26).

Adjustment for RDW quintile did not attenuate the risk associated with lymphopenia (age-, sex-, and RDW quintile–adjusted HR, 1.3 for ALC ≤1500/μL; 95% CI, 1.2-1.4). Instead, Cox proportional hazards regression models adjusting for age and sex showed evidence of interaction (*P* = .002) between the risk associated with ALC and RDW quintiles. The risk of lymphopenia occurring at higher levels of RDW is also apparent by Kaplan-Meier survival curves (eFigure 2 in the Supplement). Similarly, the risk associated with lymphopenia was not changed with additional adjustment for CRP level (age-, sex-, and CRP level quintile–adjusted HRs, 1.3 for ALC 1100/μL to 1500/μL [95% CI, 1.2-1.5] and 1.8 for ALC ≤1000/μL [95% CI, 1.6-2.1]). A more modest interaction was observed between CRP level and ALC quintiles (*P* = .04) favoring intensification of CRP level–related risk in those with lymphopenia (eFigure 3 in the Supplement). No interaction (*P* = .67) was observed between the risk associated with CRP level and RDW quintiles. In sensitivity analyses, the hazards associated with ALC, RDW, and CRP level did not diminish with exclusion of individuals with preexisting cardiovascular disease or malignant disease.

### Mortality Risk Associated With Lymphopenia and IH Dysfunction and Clinical Risk Factors

We next sought to adjust for clinical features, including age, sex, and risk factors for cardiovascular disease and malignant disease, accounting for the possibility that the association between ALC and mortality may not be consistent for all levels of age. The nonlinear interaction involving ALC and age was not statistically significant (Wald χ^2^ = 2.96; *P* = .40). However, the use of nonlinear main effects for each of these 2 variables resulted in a significantly improved model fit over simpler models that included linear ALC effects (χ^2^ = 68.0; *P* < .001) or no ALC effects altogether (χ^2^ = 85.2; *P* < .001). Red blood cell distribution width and CRP level also exhibited complex, nonlinear associations with survival. The model that incorporated tetravariate nonlinear associations involving age, ALC, RDW, and CRP level resulted in significantly improved goodness of fit over the model that only incorporated age and ALC (χ^2^ = 497.4; *P* < .001) as well as a model that included marginal (nonlinear) effects for age, ALC, RDW, and CRP level but not their interactions (χ^2^ = 65.4; *P* < .001).

After adjusting for age and other clinical risk factors, the covariate-adjusted survival curves, separately presented for each decade of age at study baseline in eFigure 4 in the Supplement, suggest that lymphopenia is independently associated with shortened survival regardless of age decade. In addition, in these fully adjusted models, a U-shaped association between levels of ALC and mortality risk emerges, suggesting expanded lymphocyte levels may also be associated with risk. Compared with the minimum risk observed at ALC levels of approximately 2000/μL, ALC levels of 1000/μL, 3000/μL, and 4000/μL had covariate-adjusted HRs of 1.6 (95% CI, 1.5-1.8), 1.1 (95% CI, 1.0-1.2), and 1.4 (95% CI, 1.2-1.8), respectively.

**Figure 3.**
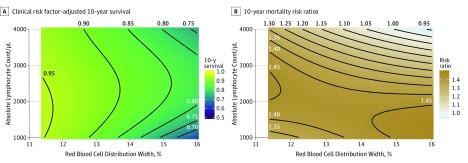
Mortality Risk According to Multiple-Parameter Immunohematologic Status Multivariable Cox proportional hazards regression models were used to depict the 10-year survival proportion of an individual aged 60 years as a function of traditional clinical risk factors as well as absolute lymphocyte count (ALC), red blood cell distribution width (RDW), and C-reactive protein (CRP) level. Contours are shown according to immunohematologic variables adjusted to represent median values of continuous covariates (systolic blood pressure: 122 mm Hg; diastolic blood pressure: 70 mm Hg; total cholesterol level: 197 mg/dL; high-density lipoprotein cholesterol level: 50 mg/dL [to convert cholesterol to millimoles per liter, multiply by 0.0259]) as well as the discrete variables of female sex, nonsmoking, no diabetes, non-Hispanic ethnicity, and white race. A, Contours of clinical risk factor–adjusted 10-year survival are shown according to RDW and ALC, centered for a CRP level of 0.5 mg/L (to convert to nanomoles per liter, multiply by 9.524). Similar survival contours are observed for other CRP levels and/or participants centered at 80 years of age as shown in eFigure 5 in the Supplement. B, Model estimates of 10-year mortality risk ratios comparing different levels of CRP (0.5 mg/L vs 0.1 mg/L) for an individual aged 60 years across the continuum of ALC and RDW. Similar survival contours are observed for participants centered at 80 years of age and for a comparison of CRP levels of 1.0 vs 0.5 mg/L as shown in eFigure 6 in the Supplement. To convert ALC to ×10^9^ per liter, multiply by 0.001.

Given the complexity of the associations among ALC, RDW, and CRP level in age- and sex-adjusted Cox proportional hazards regression models and Kaplan-Meier analyses, we next sought to analyze these associations in fully adjusted models producing contours of risk according to ALC and RDW in 2-dimensional space (Figure 3 and eFigures 5 and 6 in the Supplement). The covariate-adjusted associations between IH variables and survival arising from these models were analyzed at fixed values of age and CRP level. For instance, covariate-adjusted estimates of 10-year survival as a function of ALC and RDW for 60 years of age and CRP level of 0.5 mg/L (to convert to nanomoles per liter, multiply by 9.524) are shown in Figure 3A. Low and high ALCs were associated with decreased survival, and these curvilinear contours became more acute at higher RDW levels. For instance, a typical person aged 60 years at low risk by traditional risk factors had a 10-year survival approaching 95%, assuming an RDW of 12%, nearly irrespective of lymphocyte level. However, this same individual with an RDW of 15.0 had a survival rate of 85% with optimal lymphocyte levels and only 75% if accompanied by lymphopenia or lymphocytosis. Models centered at higher or lower CRP levels for persons aged 80 years yielded similar results (eFigure 5 in the Supplement).

We next sought to analyze the extent to which the risk associated with CRP level depended on the IH status as reflected by ALC and RDW. The covariate-adjusted risk ratios associated with CRP levels (0.5 vs 0.1 mg/L) are shown for persons aged 60 years across the landscape of ALC and RDW (Figure 3B). Elevated CRP level was associated with increased mortality risk, and this attributable risk increased in those with lower ALC (HR, approximately 1.4 for ALC of 1000/μL to 2000/μL compared with those with relative lymphocytosis (HR of approximately 1.2 for ALC of 3000/μL to 4000/μL). An analysis centered at 80 years of age and comparisons of higher CRP levels showed similar results (eFigure 6 in the Supplement).

### Multiple-Parameter Immune Status and Survival

**Figure 4.**
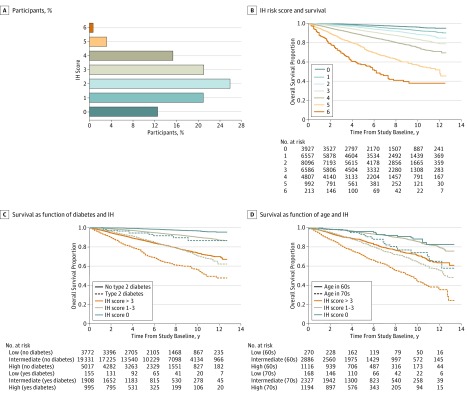
Stratification of High-Risk Populations by Multiple-Parameter Immunohematologic (IH) Status A simple IH status scoring system was used in which 1 or 2 points were assigned for mild (>1000/μL to ≤1500/μL) or severe (≤1000/μL) lymphopenia, respectively, and 1 or 2 points were assigned to participants who were in the middle or upper tertile, respectively, for red blood cell distribution width (RDW) and C-reactive protein (CRP) level. A, The percentage of participants with IH risk scores of 0 through 6 are shown. B, Kaplan-Meier curves depict the association between IH risk score and survival. C, Kaplan-Meier curves depict survival as a function of type 2 diabetes and IH status. Participants with low (IH score, 0), intermediate (IH score, 1-3), and high (IH score, >3) IH risk are shown in the presence or absence of diabetes. D, Kaplan-Meier curves show survival as a function of age and IH status. Participants with low (IH score, 0), intermediate (IH score, 1-3), or high (IH score, 4-6) IH risk profiles are shown in individuals in their 60s (mean age, 65 years) vs 70s (mean age, 75 years) at enrollment. To convert ALC to ×10^9^ per liter, multiply by 0.001.

To simplify and contextualize this aggregate IH risk, we used a simple scoring system to categorize participants according to lymphopenia status, RDW, and CRP level. Individuals who did not have lymphopenia and were in the lowest tertile for RDW and CRP level received a score of 0. Participants with mild lymphopenia (ALC>1000/μL and ≤1500/μL) received 1 point, whereas those with severe lymphopenia (ALC≤1000/μL) received 2 points. Participants in the middle and upper tertiles were assigned 1 and 2 points, respectively, for each abnormal RDW value and CRP level. In this way, individuals were stratified with IH status scores of 0 to 6, which was associated with a range of 10-year mortality rates of 3.8% to 62.1% (Figure 4A-B). Participants without any IH risk factors constituted 12.6% of the general population and had a 10-year mortality rate of only 4.0%. Most individuals (68.1%) had IH status scores from 1 to 3, placing them at intermediate IH risk, and a 10-year mortality rate of approximately 11.3%. A higher risk IH profile (IH status scores of 4-6) was nearly twice as common as prevalent type 2 diabetes (19.3% vs 10.0%) and was associated with a 3-fold elevation in age- and sex-adjusted mortality risk (HR, 3.2; 95% CI, 2.6-4.0). Those at the highest risk stratum (IH status score of 6) represented a small fraction of the population (0.7%) but had a 10-year mortality rate (62.0%) worse than that of most advanced cancers. Immunohematologic risk was additive to clinical risk. Participants with type 2 diabetes and a low-risk IH status had survival (86.6%) on par with participants without diabetes and at intermediate IH risk (90.0%) (Figure 4C). Individuals in their eighth decade of life (mean age, 75 years) with a low IH risk profile had a better prognosis than those in their 60s (mean age, 65 years) at high IH risk (IH status score, 4-6) (Figure 4D). Individuals aged 70-79 years (mean age, 75 years) with low IH risk had a 10-year survival rate of 74.1% compared with a 10-year survival rate of 68.9% for those who were a decade younger (mean age, 65 years) but with a high IH risk profile.

## Discussion

This study sought to (1) determine the associations among lymphocyte levels, other IH parameters (RDW and CRP level), and survival and (2) establish the extent to which the associated risk of these variables is additive. In this large cohort of adults, we found that lymphopenia was associated with mortality risk independently of traditional clinical risk factors and other IH variables (RDW and CRP level). Individuals with multiple IH abnormalities had a strikingly high risk of mortality among this generally low-risk population. The risk associated with this IH pattern is independent of (and thus additive to) traditional clinical risk factors. Together, these data suggest that IH risk may be viewed as a multidimensional entity and can be estimated using markers commonly available as part of routine clinical care.

### Lymphocyte Levels and Longevity in the General Population

We believe the results presented herein add to the growing body of evidence that immune status is associated with cardiovascular and noncardiovascular disease. Previous observational and prospective trials suggest that participants with overt or subacute inflammatory diseases have elevated risk of atherothrombotic disease,^36,37^ heart failure,^38,39^ malignant disease,^40^ and death.^41^ Comparatively few studies have evaluated ALC as a prognostic biomarker. Herein, we found that lymphopenia is relatively common in the general population and is associated with reduced longevity independently of age, clinical risk factors, and other IH parameters. In our fully adjusted analyses, a bimodal relationship between lymphocyte counts and mortality emerged, suggesting that the expansion of lymphocytes may also introduce hazard in the general population.

Because mortality in this population was largely driven by noninfectious causes, these data support the notion that immune status is indeed associated with resilience against cancer and cardiovascular disease, and an adverse immune phenotype may precede clinical manifestations of these illnesses. Whether lymphocyte levels are themselves part of the causal pathways linking lymphopenia to death will require additional study. Cytotoxic T cells can eradicate cells with malignant potential, and thus an optimal ALC may reflect an immune system capable of providing protection against tumor development. Lymphopenia can also induce compensatory proliferation of antigen-experienced T cells,^42^ which could increase the risk of cardiovascular disease. In those with lymphocytosis, dysregulated expansion of memory T cells, whether driven by indolent viral infections (eg, cytomegalovirus) or other mechanisms, may induce a proinflammatory milieu and similarly elevate the risk of incident cardiovascular disease.^43^

Lymphopenia may also reflect adverse inflammatory, metabolic, or neuroendocrine stressors and thus be associated with survival as an epiphenomenon. The administration of tumor necrosis factor, interleukin 1β, or microbial products reduces levels of circulating T cells. Excess levels of cortisol and catecholamine also cause lymphopenia.^26,27,28,29,30^ In these disease and models systems, lymphopenia was caused by redistribution of T cells from the circulation to lymphoid tissues^30,44,45^ and an increased susceptibility of T cells to apoptosis.^45,46^ Thus, additional study is needed to characterize the immune, metabolic, and neuroendocrine profiles in those with dysregulated T-cell homeostasis and to explore the lines of causation and effect that may contribute to resilience and longevity in the primary prevention setting.

### Multiple-Parameter Immune Status

In those participants without established inflammatory comorbidities, the use of CRP level as a marker for inflammatory risk in the general population is now well established and is associated with adverse outcomes and response to therapy independently of clinical factors.^19,20^ Other parameters of immune activation, including interleukin 6, tumor necrosis factor, and interleukin 1β, are also associated with disease risk, but the extent to which these markers represent separate vs overlapping upstream mechanisms is incompletely understood.^47,48,49,50^ Multiple groups, including our own, have identified relatively strong associations between RDW and clinical outcomes in a variety of populations.^51,52,53^ An elevated RDW may reflect chronic inflammation, ultimately leading to altered iron homeostasis and erythropoietin resistence.^54,55^ Associations between RDW and interleukin 6 have been demonstrated in heart failure^56^ and in participants with human immunodeficiency virus infection,^57^ and RDW was associated with tumor necrosis factor in those with sepsis.^58^

This is, to our knowledge, the first description of the complex associations among ALC, RDW, CRP level, and survival in the general population. We found that the risks associated with these abnormal IH parameters are not only independent of, but possibly synergistic with, each other. This observation could be consistent with a conceptual model in which an adverse inflammatory status, when severe and/or protracted, leads to end-organ effects, such as anisocytosis and/or lymphopenia, before disease incidence. Thus, the elevated risk associated with multiple IH abnormalities may reflect a more severe immune phenotype, and if so, our data suggest these high-risk participants can be identified before the clinical onset of disease by closer examination of routine blood indices. Alternatively, the mechanisms driving these IH abnormalities may be multiple hits affecting immune, neuroendocrine, and/or hematologic function, which together impair resilience against disease. Additional studies are therefore needed to ultimately determine the drivers of IH dysfunction and its role in survival among the general public.

### Limitations

This study is limited by its observational design. Although the NHANES cohort represents a large sample in which we were able to use rigorous biostatistical techniques, we cannot exclude residual confounding, especially by unmeasured variables. We assessed death by linking vital status through the National Death Index to NHANES participants. Because such linkage is incomplete, this could introduce bias. Also, specific cause of death was not available, and therefore additional studies are needed to determine whether IH associations hold for specific cancers, specific forms of cardiovascular diseases, and/or specific infections. Whether these associations hold for hospitalized patients also deserves additional study. As with all observational studies, the extent to which these associations may be causal in nature could not be assessed.

## Conclusions

Approximately 20% of the general US population appears to have a high-risk IH profile, and these participants’ 10-year mortality was 28%, compared with 4% in participants in the present study with a low-risk profile. Moreover, in this cohort study, those individuals with important clinical risk factors who had an adverse IH status were at extreme risk of mortality. Because the IH profiles defined herein emerge from routine testing, ensuring that these high- and extreme-risk individuals receive appropriate evidence-based disease preventive services (cancer screening, vaccination, lifestyle changes, and cardiovascular risk factor modification) may be a pragmatic, simple way to improve the effectiveness and efficiency of population health efforts.

## Notes

Supplement. **eFigure 1.** Multiple Immunohematologic Variables Associated With Survival **eFigure 2.** Risk Associated With Lymphopenia Accentuated When Coexistent With Anisocytosis **eFigure 3.** Risk Associated With CRP Within Various Lymphocyte Strata **eFigure 4.** Lymphocyte Levels Independently Associated With Survival in Each Decade of Age **eFigure 5.** Mortality Risk According Immunohematologic Status at Various CRP Levels and Ages **eFigure 6.** Mortality Risk Associated With CRP Across the Immunohematologic Landscape at Various CRP Levels and Ages **eTable.** Correlations Between Immune and Hematologic Indices
