# Supplementary material for: Association of Lymphopenia With Risk of Mortality Among Adults in the US General Population
Source: JAMA Netw Open. 2019 Dec 2;2(12):e1916526. doi: 10.1001/jamanetworkopen.2019.16526 (PMC6902755; doi:10.1001/jamanetworkopen.2019.16526)
Supplement: Supplement. — eFigure 1. Multiple Immunohematologic Variables Associated With Survival eFigure 2. Risk Associated With Lymphopenia Accentuated When Coexistent With Anisocytosis eFigure 3. Risk Associated With CRP Within Various Lymphocyte Strata eFigure 4. Lymphocyte Levels Independently Associated With Survival in Each Decade of Age eFigure 5. Mortality Risk According Immunohematologic Status at Various CRP Levels and Ages eFigure 6. Mortality Risk Associated With CRP Across the Immunohematologic Landscape at Various CRP Levels and Ages eTable. Correlations Between Immune and Hematologic Indices [file jamanetwopen-2-e1916526-s001.pdf]

Supplementary Online Content

Zidar DA, Al-Kindi SG, Liu Y, et al. Association of lymphopenia with risk of mortality among adults in the US general population. *JAMA Netw Open.* 2019;2(12):e1916526. doi:10.1001/jamanetworkopen.2019.16526

eFigure 1. Multiple Immunohematologic Variables Associated With Survival

eFigure 2. Risk Associated with Lymphopenia Accentuated When Coexistent With Anisocytosis

eFigure 3. Risk Associated With CRP Within Various Lymphocyte Strata

eFigure 4. Lymphocyte Levels Independently Associated With Survival in Each Decade of Age

eFigure 5. Mortality Risk According Immunohematologic Status at Various CRP Levels and Ages

eFigure 6. Mortality Risk Associated With CRP Across the Immunohematologic Landscape at Various CRP Levels and Ages

eTable. Correlations Between Immune and Hematologic Indices

This supplementary material has been provided by the authors to give readers additional information about their work.

© 2019 Zidar DA et al. *JAMA Network Open.*

eFigure 1. Multiple Immunohematologic Variables Associated With Survival

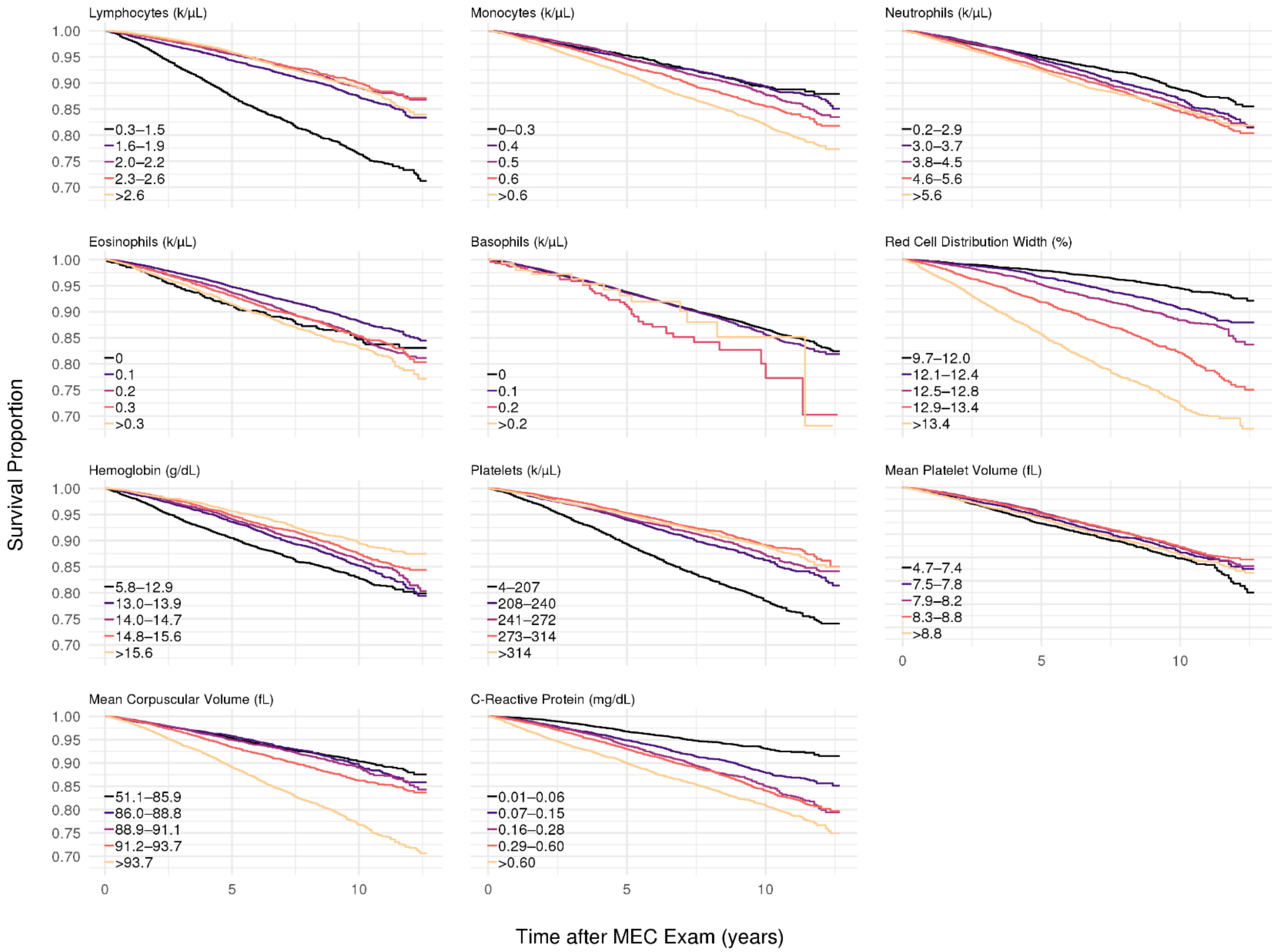

Univariable Kaplan-Meier estimates of survival are shown, stratified according to quartiles of common immuno-hematologic and inflammatory biomarkers.

© 2019 Zidar DA et al. *JAMA Network Open.*

eFigure 2. Risk Associated with Lymphopenia Accentuated When Coexistent With Anisocytosis

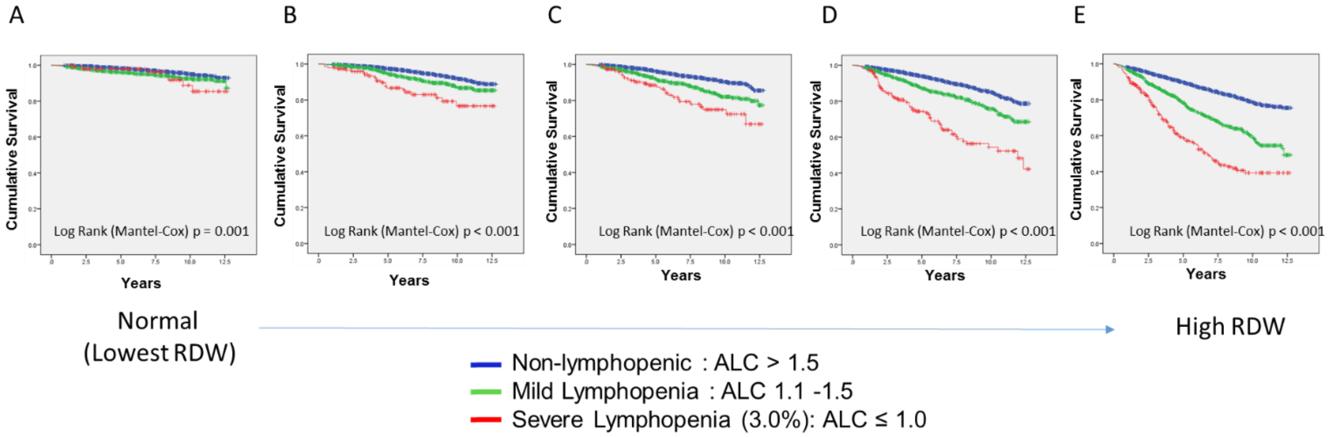

Survival according to lymphopenia status across RDW strata is determined using Kaplan Meier curves. Participants without lymphopenia (absolute lymphocyte count, ALC > 1.5 cells/µL, blue) are compared to those with modest (ALC >1.0, ≤ 1.5 cells/µL, green) and severe (ALC ≤ 1.0 cells/µL, red) lymphopenia, grouped according to red cell distribution n width (RDW) quintiles ranging from the (**A**) lowest (≤ 12.0), (**B**) 2nd quintile (12.1-12.4), (**C**) 3rd quintile (12.5- 12.8), (**D**) 4th quintile (12.9-13.4), and (**E**) highest quintile (≥ 13.5).

© 2019 Zidar DA et al. *JAMA Network Open.*

eFigure 3. Risk Associated With CRP Within Various Lymphocyte Strata

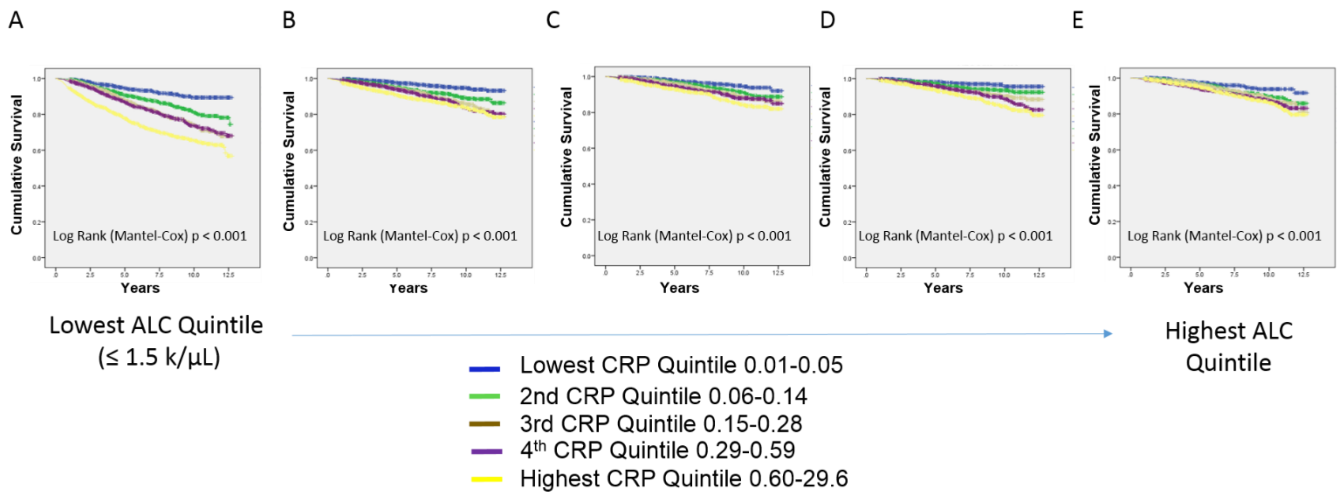

Kaplan Meier curves demonstrate survival according to CRP across lymphocyte quintiles. Participants with various levels of CRP (quintiles) are analyzed among those with lymphopenia (**A**: lowest ALC quintile, ALC ≤ 1.5 cells/μL), and increasing quintiles of ALC (**B-E**).

© 2019 Zidar DA et al. *JAMA Network Open.*

eFigure 4. Lymphocyte Levels Independently Associated With Survival in Each Decade of Age

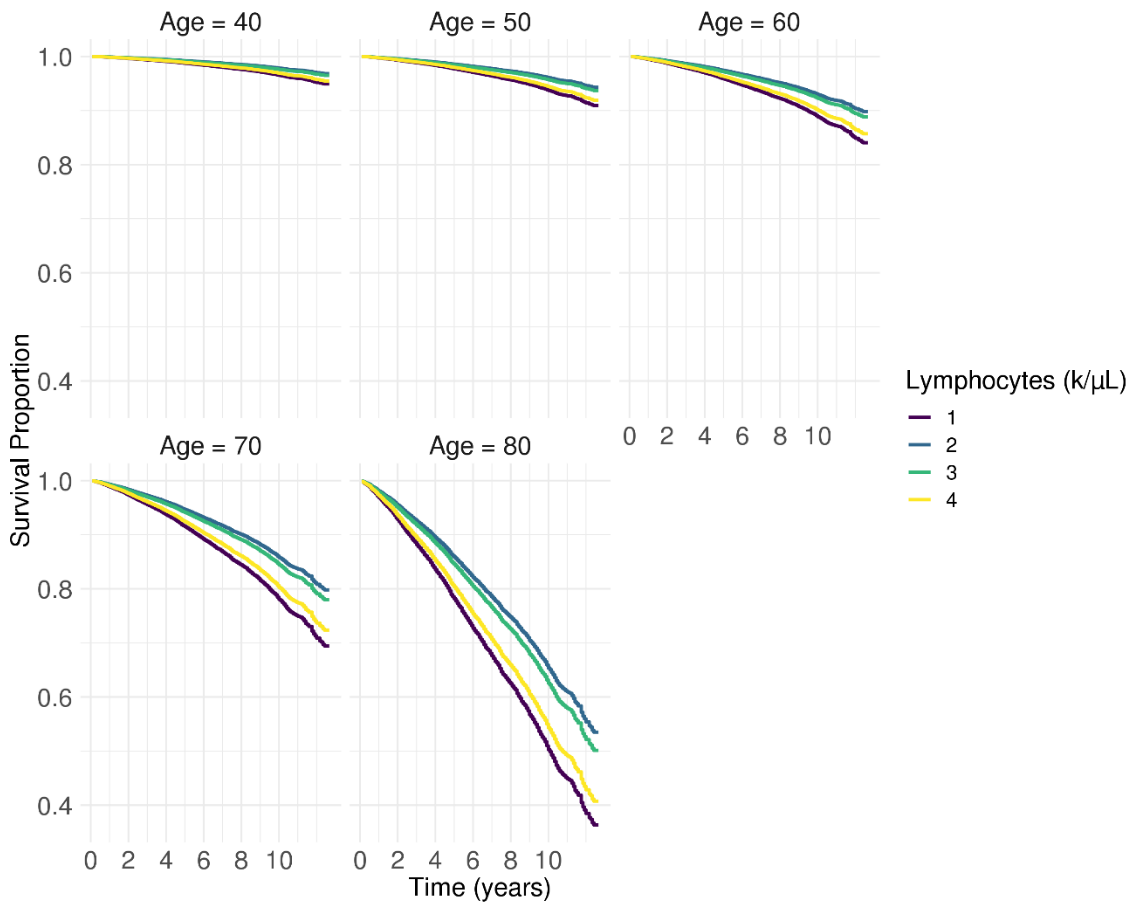

Multivariable Cox proportional hazards regression models were used to generate covariate-adjusted survival curves after study baseline for different levels of ALC at study baseline. Separate panels are presented for each decade of age. Curves are adjusted to represent median values of continuous covariates (systolic blood pressure: 122 mmHg; diastolic blood pressure: 70 mmHg; total cholesterol: 197 mg/dL; high-density lipoprotein cholesterol: 50 mg/dL) and discrete patient characteristics (female sex, non-smoker, non-diabetic, non-Hispanic ethnicity, and Caucasian race).

© 2019 Zidar DA et al. *JAMA Network Open.*

eFigure 5. Mortality Risk According Immunohematologic Status at Various CRP Levels and Ages

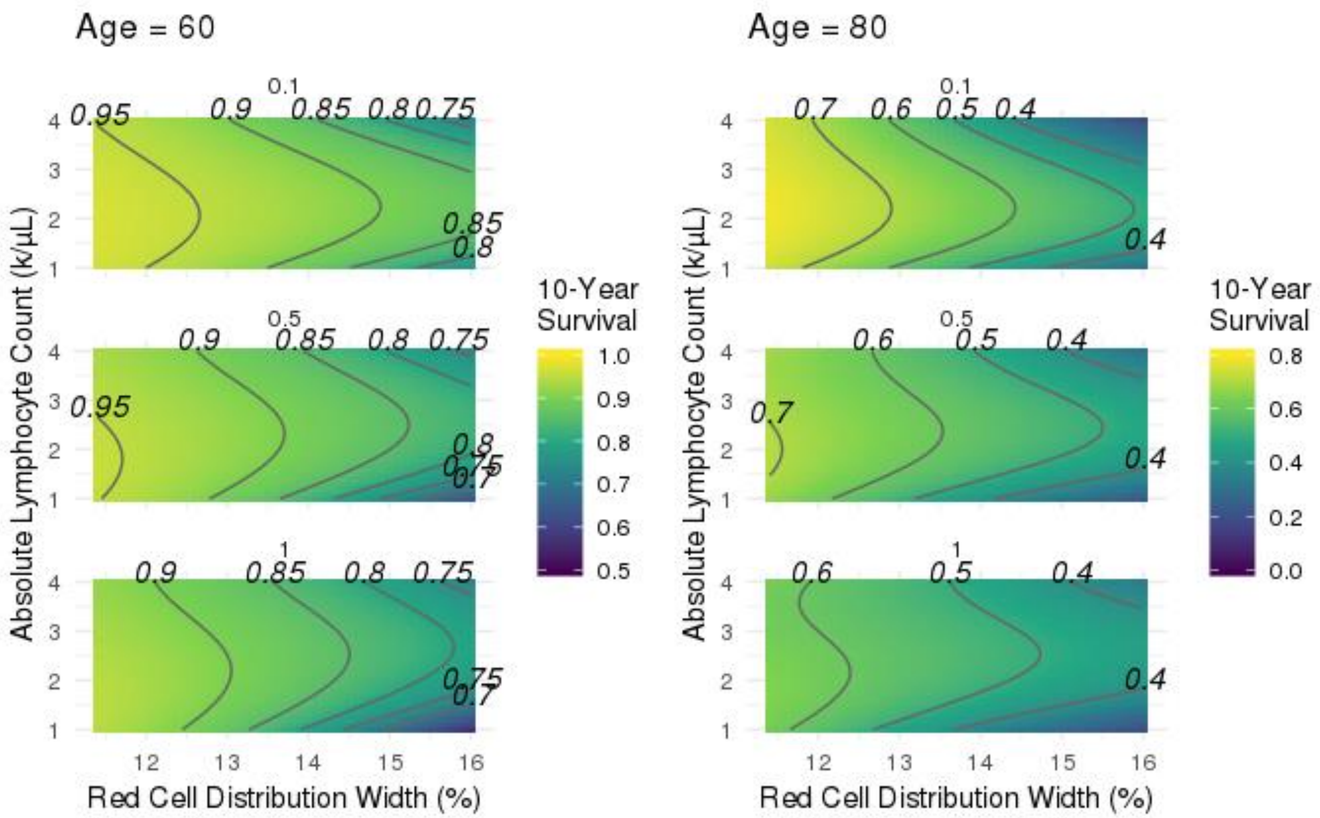

Multivariable Cox proportional hazards regression model were used to depict 10-year survival proportion according to ALC and RDW. Contours according to these immunohematologic variables were derived after adjustment for systolic blood pressure, diastolic blood pressure, total cholesterol, high-density lipoprotein cholesterol, sex, smoking status, diabetes, ethnicity, and race at age centered at 60 (left panels) and 80 years (right panels), and at CRP centered at 0.1 (top panels), 0.5 (middle panels), and 1.0 (lower panels).

© 2019 Zidar DA et al. *JAMA Network Open.*

eFigure 6. Mortality Risk Associated With CRP Across the Immunohematologic Landscape at Various CRP Levels and Ages

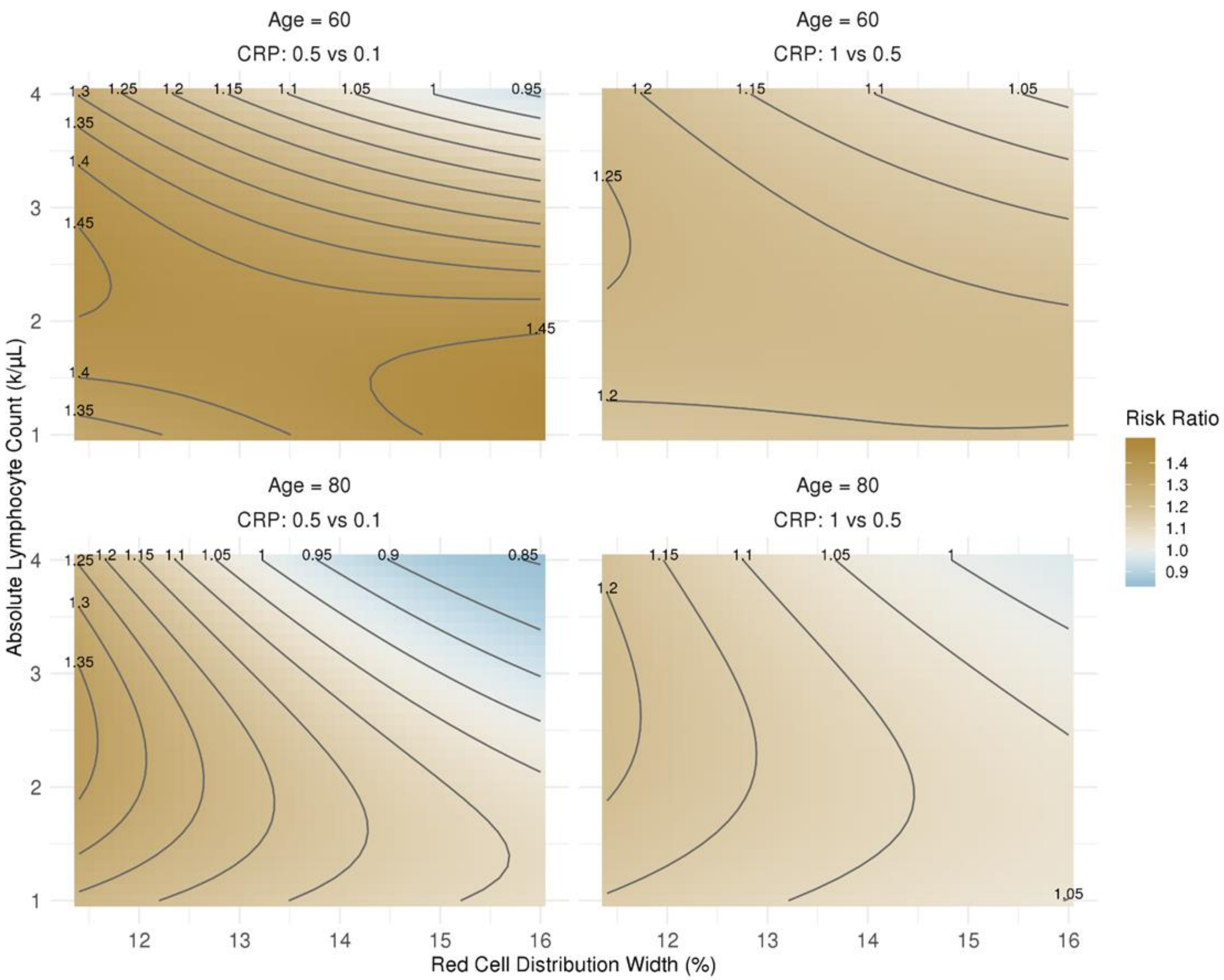

Multivariable Cox proportional hazards regression model were used to depict the risk ratio of different CRP levels according to ALC and RDW positioning. Contours were derived after adjustment for systolic blood pressure, diastolic blood pressure, total cholesterol, high-density lipoprotein cholesterol, sex, smoking status, diabetes, ethnicity, and race at age centered at 60 (top panels) and 80 years (bottom panels), for CRP comparisons between 0.5 versus 0.1 mg/L (leftward panels) and 1.0 versus 0.5 mg/dL (rightward panels).

© 2019 Zidar DA et al. *JAMA Network Open.*

eTable. Correlations Between Immune and Hematologic Indices

Spearman Correlation Coefficient [95% Confidence										
	AMC2	ANC3	AEC4	ABC5	RDW6	HGB7	PLT8	MPV9	MCV10	CRP11
ALC1	0.30 [0.28, 0.31]	0.19 [0.18, 0.20]	0.20 [0.19, 0.21]	0.22 [0.21, 0.23]	-0.04 [-0.05, -0.03]	0.05 [0.04, 0.06]	0.25 [0.24, 0.26]	0.02 [0.01, 0.03]	-0.08 [-0.09, -0.07]	0.07 [0.06, 0.08]
AMC2		0.40 [0.39, 0.41]	0.23 [0.22, 0.24]	0.19 [0.17, 0.20]	0.03 [0.02, 0.04]	0.11 [0.10, 0.12]	0.11 [0.10, 0.13]	0.02 [0.01, 0.03]	0.05 [0.04, 0.06]	0.14 [0.12, 0.15]
ANC3			0.14 [0.13, 0.15]	0.23 [0.22, 0.24]	0.05 [0.03, 0.06]	0.03 [0.02, 0.04]	0.22 [0.21, 0.23]	0.03 [0.02, 0.04]	-0.02 [-0.03, -0.01]	0.29 [0.28, 0.30]
AEC4				0.19 [0.18, 0.20]	0.02 [0.01, 0.03]	0.10 [0.09, 0.11]	0.09 [0.08, 0.10]	0.01 [0.00, 0.02]	-0.01 [-0.02, 0.00]	0.09 [0.08, 0.10]
ABC5					0.03 [0.02, 0.04]	-0.02 [-0.03, -0.01]	0.15 [0.14, 0.16]	0.01 [0.00, 0.02]	-0.03 [-0.05, -0.02]	0.09 [0.08, 0.10]
RDW6						-0.29 [-0.30, -0.28]	0.03 [0.02, 0.04]	0.02 [0.01, 0.03]	-0.28 [-0.29, -0.26]	0.23 [0.21, 0.24]
HGB7							-0.12 [-0.14, -0.11]	0.02 [0.01, 0.04]	0.20 [0.19, 0.21]	-0.19 [-0.20, -0.18]
PLT8								-0.36 [-0.37, -0.35]	-0.16 [-0.17, -0.15]	0.18 [0.17, 0.19]
MPV9									-0.02 [-0.04, -0.01]	0.01 [0.00, 0.02]
MCV10										-0.12 [-0.13, -0.11]

1 ALC = Absolute Lymphocyte Count; 2 AMC = Absolute Monocyte Count; 3 ANC = Absolute Neutrophil Count; 4 AEC = Absolute Eosinophil Count; 5 ABC = Absolute Basophil Count; 6 RDW = Red Cell Distribution Width; 7 HGB = Hemoglobin; 8 PLT = Platelet Count; 9 MPV = Mean Platelet Volume; 10 MCV = Mean Corpuscular Volume; 11 CRP = C-reactive Protein

© 2019 Zidar DA et al. *JAMA Network Open.*
